# Retraction Note to: Exosome-mediated lncRNA AFAP1-AS1 promotes trastuzumab resistance through binding with AUF1 and activating ERBB2 translation

**DOI:** 10.1186/s12943-022-01610-z

**Published:** 2022-07-11

**Authors:** Mingli Han, Yuanting Gu, Pengwei Lu, Jingyi Li, Hui Cao, Xiangke Li, Xueke Qian, Chao Yu, Yunqing Yang, Xue Yang, Na Han, Dongwei Dou, Jianguo Hu, Huaying Dong

**Affiliations:** 1 Department of Breast Surgery, The First Affiliated Hospital of Zhengzhou University, Zhengzhou, 450052 China; 2 Department of Vascular Surgery, The First Affiliated Hospital of Zhengzhou University, Zhengzhou, 450052 China; 3 Department of Oncology, The First Affiliated Hospital of Zhengzhou University, Zhengzhou, 450052 China; 4 Department of General Surgery, University-Town Hospital of Chongqing Medical University, Chongqing, 400016 China; 5 Department of Obstetrics and Gynecology, The Second Affiliated Hospital, Chongqing Medical University, Chongqing, 400010 China; 6 Department of General Surgery, Hainan General Hospital, Hainan Affiliated Hospital of Hainan Medical University, No.19 XiuHua Road, Xiuying District, Haikou, 570311 China

**Retraction note to: Mol Cancer 19, 26 (2020)**

**https://doi.org/10.1186/s12943-020-1145-5**

The Editor-in-Chief has retracted this article. After publication, concerns were raised regarding suspected image overlap with three other articles [1–3]. Specifically, in Fig. 2d, all subpanels appear to be highly similar to the images in Fig. 3c (rotated) in [1]. In Fig. 2f, a number of images appear highly similar to those in Figs. 5a and c in ([2], now retracted) and 3e in [3]. In addition, Fig. 5e appears to be drawn by hand.

The authors have provided the original data and revised figures to address the concerns. However, the original data contains highly similar backgrounds in the western blot gel photographs. Additionally, the source, number and sex of animals are different between the Ethics approval document and the Methods section of the article.

The Editor-in-Chief therefore no longer has confidence in the presented data and the ethical standards of the study.

Hui Cao agrees to this retraction. Mingli Han and Huaying Dong have stated on behalf of all co-authors that they agree to this retraction.
